# Carbon load in airway macrophages as a biomarker of exposure to particulate air pollution; a longitudinal study of an international Panel

**DOI:** 10.1186/s12989-018-0250-8

**Published:** 2018-03-14

**Authors:** Yang Bai, Hannelore Bové, Tim S. Nawrot, Benoit Nemery

**Affiliations:** 1 Centre for Environment and Health, Department of Public Health and Primary Care, KU Leuven, Herestraat 49, O&N 1, box 706, 3000 Leuven, Belgium; 2 Biomedical Research Institute, Hasselt University, Agoralaan Building C, 3590 Diepenbeek, Belgium; 3 Center for Surface Chemistry and Catalysis, KU Leuven, Celestijnenlaan 200F, 3001 Leuven, Belgium; 4 Centre for Environmental Sciences, Hasselt University, Agoralaan Building D, 3590 Diepenbeek, Belgium

**Keywords:** Carbon load, Airway macrophages, Internal biomarker, Air pollution, Exposure assessment, Lung clearance, Kinetic behavior, Global health

## Abstract

**Background:**

Carbon load in airway macrophages (AM) has been proposed as an internal marker to assess long-term exposure to combustion-derived pollutant particles. However, it is not known how this biomarker is affected by changes in exposure. We studied the clearance kinetics of black carbon (BC) in AM, obtained by sputum induction, in a one-year panel study.

**Methods:**

AM BC was measured 8 times with 6 weeks intervals in healthy young subjects: 15 long-term residents in Leuven, Belgium (BE, mean annual PM_10_ 20–30 μg/m^3^) and 30 newcomers having arrived recently (< 3 weeks) in Leuven from highly polluted cities (mean annual PM_10_ > 50 μg/m^3^) in low and middle-income countries (LMIC, *n* = 15), or from low to moderately polluted cities in high-income countries (HIC, n = 15). The median and 90th percentile values of AM BC were quantified by image analysis of 25 macrophages per sputum sample; the carbonaceous nature of the black inclusions in AM was verified by Femtosecond Pulsed Laser Microscopy in 30 macrophages. We used a Bayesian hierarchical single-exponential decay model to describe the evolution of AM BC.

**Results:**

In the LMIC group, the mean (95% credible interval) initial quantity (R_0_) of median AM BC [1.122 (0.750–1.509) μm^2^] was higher than in the HIC group [0.387 (0.168–0.613) μm^2^] and BE group [0.275 (0.147–0.404) μm^2^]. Median AM BC content decreased in the LMIC group (decay constant 0.013 μm^2^/day), but remained stable over one year in the other two groups. In the LMIC group, clearance half-lives of 53 (30–99) and 116 (63–231) days, were calculated for median and 90th percentile AM BC, respectively.

**Conclusions:**

In this real-life study of an international panel of healthy young subjects, we demonstrated that carbon load in airway macrophages obtained by induced sputum reflects past long-term exposure to particulate air pollution. Values of AM BC do not change over one year when exposure remains stable, but AM BC decreases upon moving from high to moderate exposure, with average half-lives of 53 and 116 days depending on the carbon load.

**Electronic supplementary material:**

The online version of this article (10.1186/s12989-018-0250-8) contains supplementary material, which is available to authorized users.

## Background

Assessing individual exposure to airborne pollutants is of critical importance in toxicological and epidemiological studies. In the early studies, individual exposure to air pollution was generally inferred from measurements made by monitoring stations, under the ecological assumption that all persons living in a certain area shared the same exposure. More recently, improvements in assessing (short-term) temporal and spatial variations in exposure have been achieved with the development of portable monitoring devices [1] and the utilization of geographic information systems [1–4]. Nevertheless, these techniques are based on estimating or measuring *external* exposure, which does not necessarily equate to measuring the amounts of air pollutants that have been actually inhaled by individuals over a prolonged time course.

An alternative to estimating external exposures consists in biomonitoring, i.e. measuring concentrations of pollutants (or their metabolites) in individual subjects, usually in urine or blood [2]. Biomonitoring has been utilized to a limited extent and with limited success for assessing chronic exposure to combustion-related chemicals [5]. However, a promising approach to assess long-term exposure to traffic-related particulate pollution consists in measuring black carbon (BC) content in airway macrophages (AM), which can be obtained by inducing sputum in children or adults [6]. Various studies have demonstrated that the surface occupied by black inclusions in AM is indicative of the intensity of exposure to elemental carbon derived from the combustion of fossil fuels [7, 8] or from the burning of biomass [9]. In other words, AM BC directly reflects the *internal* concentration of inhaled particles and, therefore, mirrors variations in personal exposure to environmental particulate air pollution.

Nevertheless, as concluded in our review on the subject [6], several questions still remain unresolved with regard to measuring carbon load in AM to assess long-term exposure to traffic-related air pollution. One important issue that has not been adequately evaluated concerns the time window of past exposure to air pollution that is reflected by the amount of carbon found in airway macrophages, i.e. the kinetic behavior of AM BC retrieved by induced sputum has not been assessed.

The majority of particles that have been deposited in the alveoli are phagocytosed by AM and, assuming a one-compartment clearance model [10], these AM are eliminated from the lungs with a certain clearance constant (k). We hypothesized that individuals moving from a country with high ambient air pollution to a country with less pollution would exhibit a high BC load in AM upon arrival and their AM BC would progressively decrease from an initial quantity (R_0_) to become similar to that of individuals having resided for a long time in the destination area. Conversely, individuals moving from an area of lower pollution would exhibit a progressive increase in AM BC. So, we made repeated measurements of AM BC over nearly one year in healthy young volunteers after their translocation from countries with various degrees of ambient pollution to an area of moderate pollution (Leuven, Belgium), and compared their data with those obtained similarly from long-time Leuven residents.

## Methods

### Study design

For this quasi-experimental study, we took advantage of the presence of many international students and researchers at the University of Leuven (KU Leuven). We enrolled international students and researchers as soon as possible after their arrival in Leuven (in practice, within 3 weeks), as well as control subjects comprised of either locally born Belgian residents or international researchers having resided for at least one year in Leuven. The purpose was to have all participants undergo a series of eight clinical visits (T1 – T8), with about 6 weeks between each visit. The study protocol was approved by the Ethical Committee of KU Leuven (S55729). All participants were given detailed oral and written information on the study and gave their written informed consent.

### Participants

Between 2013 and 2015, we used advertisements on university bulletin boards, online social networks, and word of mouth to enroll potential participants. Inclusion criteria included: 1) having never smoked or having quit smoking for more than 1 year, 2) having resided in Belgium for more than 1 year (“residents”) or having arrived in Belgium less than 3 weeks before enrollment (“newcomers”), 3) having lived in the same place in the previous 3 months, and 4) being available and agreeing to participate in the repeated visits over the next year. Exclusion criteria were: 1) unclear or variable residential exposures prior to arrival in Belgium, and 2) having any severe respiratory disease, a history of cardiovascular disease, diabetes, or other conditions that could interfere with the health measurements. Participants received a small recompense (cinema ticket) at each visit.

### Study groups

We initially intended to have a control group consisting of subjects having resided in Belgium for more than one year (mean annual PM_10_ between 20 and 30 μg/m^3^) and three groups of newcomers having resided, prior to enrollment into the study, in cities or areas with different degrees of air pollution, i.e. very high exposure (PM_10_ > 50 μg/m^3^), high exposure (30 ≤ PM_10_ ≤ 50 μg/m^3^) and low exposure (PM_10_ ≤ 20 μg/m^3^), as determined by the level of annual exposure to PM_10_ according to the World Health Organization (WHO) database [11]. However, by the end of enrollment, we did not have sufficient numbers of participants in each category and we, therefore, redefined the exposure groups for the statistical analysis into the following three major study groups:

1. Group BE (control) consisting of “residents in Belgium” (20 ≤ PM_10_ ≤ 30 μg/m^3^)
2. Group LMIC consisting of “newcomers from low and middle income countries (LMIC) with high pollution” (mean annual PM_10_ > 50 μg/m^3^)
3. Group HIC consisting of “newcomers from high income countries (HIC) other than Belgium” (mean annual PM_10_ < 50 μg/m^3^)

In secondary analyses, we subdivided Group BE into a subgroup of native Belgians (BE*native*) and a subgroup of foreign-born residents (BE*foreign*), and Group HIC into subgroup HIC*moderate* (newcomers from countries with annual average PM_10_ between 20 and 50 μg/m^3^) and HIC*low* (newcomers from countries with annual average PM_10_ below 20 μg/m^3^).

### Questionnaires

To recruit suitable participants, a baseline questionnaire was distributed to obtain information on the participants’ prior residences, living environment, environmental exposures, cigarette smoke and biomass smoke exposures, whereabouts in the past 6 months, medical history, and recent incidence of cold and other respiratory diseases. At each of the following visits, another questionnaire enquired about changes in residential location, past travel outside Belgium, cigarette smoke and biomass smoke exposures, physical condition, newly acquired diseases (including colds).

### Induced sputum and measurement of carbon load in airway macrophages

Sputum induction was performed, under the supervision of the same operator (YB), in the Laboratory of Pulmonary Function Testing of the UZ Leuven, according to a standard protocol, based on international guidelines [12] (see Additional file 1 for details). In brief, the subject performed spirometry [13] both before and after inhalation of salbutamol 400 μg and, if forced expiratory volume in 1 s (FEV_1_) after salbutamol was > 80% predicted, the subject inhaled an aerosol of hypertonic saline (3%, 4%, and 5%) for successive periods of 7 min to cough up sputum. Visible plugs were treated with dithiothreitol, suspended in Hank’s balanced salt solution, cytocentrifuged and slides were stained with Diff-Quik.

The area of carbon in AM was determined as previously described [14] and without knowledge of the group or time point of obtaining the sample, because the operator (YB) randomly picked individual cytospin slides at the end phase of the project after completion of sampling. Briefly, digital images of 25 randomly selected AM from each cytospin slide were obtained at × 100 magnification. Preliminary experiments performed on previously obtained samples indicated that the carbon load estimated by assessing 25 AMs did not differ from the figure based on 50 AMs. Color images were converted to 32-bit black and white images using ImageJ (National Institutes of Health, USA). Automatic “threshold” command and freehand selection were combined to select the black particles that were within the cell. The software generated a number of pixels which was converted to an area in micrometers squared (for our analysis: 146 pixels = 10 μm at × 100 magnification). The median area (μm^2^) from 25 AM was calculated, but we also considered the 90th percentile of the distribution.

In three cytospin slides from a participant exhibiting a broad range of carbon load in the macrophages, we verified that the black inclusions detected by light microscopy in macrophages corresponded with black carbon using Femtosecond Pulsed Laser Microscopy, a method described recently by Bové et al. [15]. In brief, both two-dimensional (2D) images and three-dimensional (3D) z-stacks were acquired throughout the cells using a Zeiss LSM510 META NLO scan head mounted on an inverted laser-scanning microscope (Zeiss Axiovert 200 M; Zeiss, Germany). BC particles inside the AM were visualized using a 40×/1.1 water immersion objective and illumination by femtosecond laser pulses (810 nm, MaiTai, Spectra Physics, USA) causing them to specifically generate white light. Images were captured using the AIM 4.2 software (Carl Zeiss) and processed with the image-processing program Fiji (ImageJ v1.47, open source software, http://fiji.sc/Fiji). To display the location of BC, the “Making Montage” command in ImageJ was used directly on the 3D stacks. For quantifying the area of AM BC on the 3D stacks, “Z Project in Max Intensity” was first applied to the stacks and then converted to 32-bit black and white images. These black and white images converted from 3D stacks and the other 2D images were processed in the same way as mentioned above to obtain the areas of AM BC (for 3D converted images, 158 pixels = 41 μm; for 2D images, 75 pixels = 10 μm).

Results from spirometry and from measurements in urine and blood will be presented separately in another article.

### Data analysis

Statistical analyses were performed using R version 3.3.3 (R Foundation for Statistical Computing, Vienna, Austria) in RStudio or SAS 9.4 (SAS institute, Cary, NC, USA).

Standard descriptive statistics were used to present baseline characteristics of the study participants in different groups, and differences among groups were tested using one-way ANOVA and χ^2^ test, as appropriate. The agreement between the quantification of black inclusions by light microscopy and by Femtosecond Pulsed Laser Microscopy was tested by a Bland-Altman diagram.

The individual AM BC data obtained from each individual at each time point were used to model the time course (kinetics) of AM BC after inclusion in the study. A Bayesian hierarchical single exponential decay model (described in detail in Additional file 1) was used to estimate the initial quantity of AM BC (R_0_) and the decay constant (k). For participants belonging to the BE group, R_0_ was set as the value obtained at the first measurement (T1). For the newcomers, the first measurement did not coincide exactly with their arrival date (first measurement within 3 weeks after arrival); hence, a model-based extrapolation for the value on the arrival day (day 0) was estimated for R_0_. Two models, with or without taking the group differences into account, were compared to test for the group effect. Multiple models were used to test for the effects of covariates including gender, age, and season. The Deviance Information Criterion (DIC) was used to make relative comparisons. Additionally, posterior predictive checks were used to test if the model fitted the data well. To evaluate differences of parameters from each other, 95% credible intervals were used.

The clearance half-life for the LMIC group was calculated from the exponential decay model, as Clearance half-life = ln(2)/k.

## Results

### Participants and descriptive data

A total of 166 questionnaires were distributed to persons who had expressed interest in participating in the study. Of the 113 candidates who returned the questionnaires, 73 met the inclusion criteria and 61 participants attended the first health measurement. The data from 11 subjects who withdrew after the first (*n* = 5) or second (*n* = 6) visit, were not included in the analysis. Of the 50 remaining subjects, 43 completed all 8 visits (T1–T8), and 3, 2, and 2 completed 6 visits (T1–T6), 5 visits (T1–T5), and 4 visits (T1–T4), respectively.

In these 50 subjects, sputum induction was performed 379 times in total, with 321 samples (85%) yielding a sufficient number of AMs (≥ 25). Four participants were never able to provide suitable sputum samples, and these subjects were excluded from the analysis. In addition, we also excluded one participant (from the HIC group) with highly variable and outlying values of AM BC and whose questionnaire revealed that he had made multiple travels within and outside Europe during the observation period (the latter exclusion did not affect the overall results).

Table 1 summarizes the main characteristics, at baseline, of the 45 participants who were included in the final analysis. A greater proportion of LMIC participants were female, but the three groups did not differ in age, BMI, smoking status, and asthma. Pre-bronchodilator lung function was significantly lower in the LMIC group than in the other two groups. The LMIC participants had, by definition, a higher prior exposure to PM_10_ than the other participants. Additional information on exposures is summarized in Additional file 1: Table S1.

**Table 1:** Demographics and lung function in the 45 participants included for analysis

Group	All	LMIC	HIC	BE
Number	45	15	15	15
Age, yr	26.9 (4.9)	25.7 (3.3)	26.0 (4.9)	28.5 (5.8)
Gender, male^†^	24 (53)	6 (40)	10 (67)	8 (53)
BMI, kg/m^2^	22.8 (4.0)	21.3 (3.4)	22.9 (2.8)	24.2 (5.1)
Never/ex-smoker^†^	41/4 (91/9)	15/0 (100/0)	12/3 (80/20)	14/1 (93/7)
Mild asthma^†^	6 (13)	0	4 (27)	2 (13)
FVC, % predicted	104 (13)	93 (10)***	112 (12)	106 (12)
FEV_1_, % predicted	99 (14)	88 (13)***	109 (11)	99 (10)
Estimated annual average PM_10_ before inclusion, μg/m^3‡^		108 (63–171)	23 (14–34)	23
Latency between arrival and inclusion^‡^		9 days (1–19)	10 days (2–23)	1.9 years^#^ (1–5.4)

*LMIC* low and middle-income countries, *HIC* high-income countries, *BE* Belgium

Data are described as mean (SD) except for ^†^n (%) and ^‡^median (range). ^#^Only for 8 foreign-born residents ***p < 0.001 compared to BE group (one-way ANOVA)

In the LMIC group, 10 subjects came from China, 3 from India, and one each from Brazil and Peru. In the HIC group, 5 subjects came from Spain, 3 from Italy, 2 from the UK, and one each from The Netherlands, Croatia, Luxembourg, Ireland, and Estonia (cities of previous residence with their mean annual PM_10_ can be found in Additional file 1: Table S2). In the BE group, 7 subjects were Belgian-born residents and 8 were foreign-born (2 from Brazil, one each from Germany, Poland, Greece, Bolivia, Peru, Philippines).

### Carbon validation by femtosecond pulsed laser microscopy

**Fig. 1:**
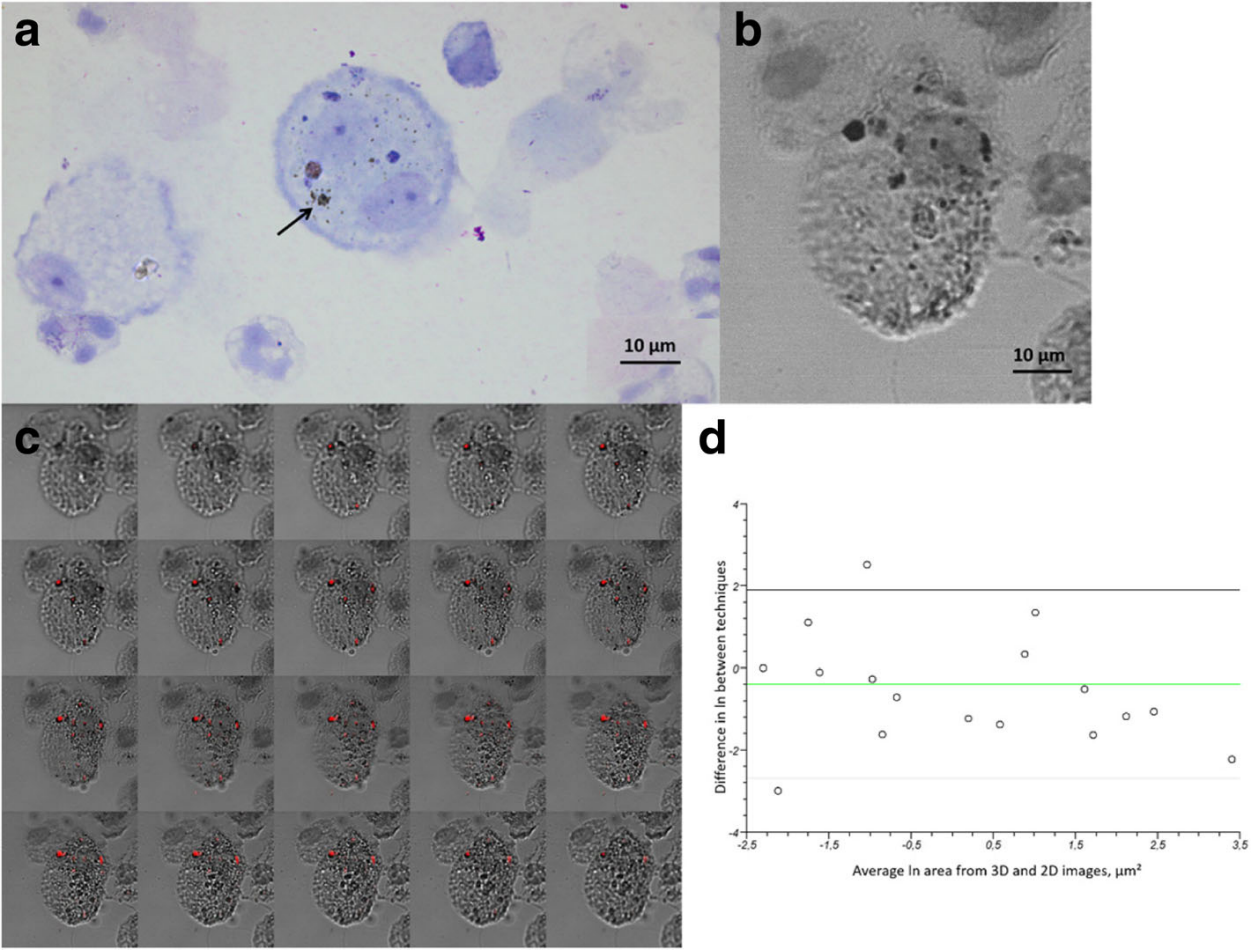
Illustration of Femtosecond Pulsed Laser Microscopy detection of BC in a macrophage. **a** Illustration of an image captured for image analysis showing two airway macrophages (AMs) stained by Diff-Quik from a participant in the LMIC group. The black arrow points out carbon in AM. Image at × 100, bar = 10 μm. **b** Light microscope (× 100) image of a single AM stained by Diff-Quik. The surface of black inclusions is used to estimate the amount of engulfed BC. **c** Montage retrieved from the same AM as the one shown in Panel b, of successive images obtained by white light 3D z-stacks scanning with 0.2 μm interval (from left top to right bottom), showing engulfed BC in red. **d** Bland-Altman plot comparing the difference between the median areas of 30 AM BC obtained from 3D converted images and 2D images. The green line represents the mean of differences in individual values (expressed as natural logarithm) obtained by the two methods; solid lines represent 2 SDs

Figure 1a shows a view of AMs, as captured for image analysis, from a participant from the LMIC group. Figure 1b and c illustrate one macrophage captured on normal 2D image and on 3D z-stacks with white light. The median (IQR) areas of AM BC obtained from 2D images and 3D converted images were 0.330 (2.855) μm^2^ and 0.482 (3.214) μm^2^, respectively. There was a positive correlation between these results (Spearman rank *r* = 0.87, 95% confidence interval: 0.72 to 0.94) and the agreement between the two techniques was high (intraclass correlation coefficient = 0.80), as assessed by Bland-Altman plot (Fig. 1d).

### Carbon load in airway macrophages

#### Time course of AM BC

**Fig. 2:**
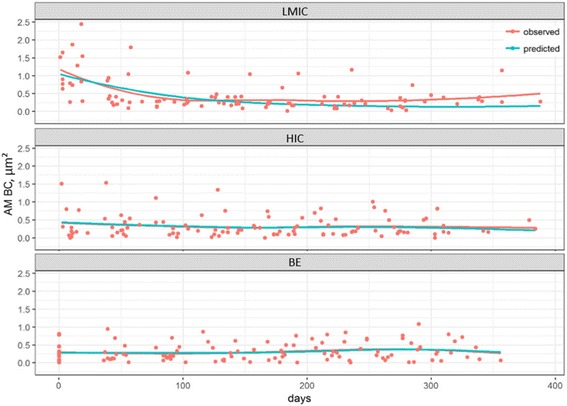
AM BC profiles with posterior predictive check for groups. Observed loess-smoothed AM BC profiles (red lines) versus model predicted loess-smoothed AM BC profiles (blue lines). Each dot represents the median value of AM BC obtained from one participant at each one of 8 time points (15 participants in each group). LMIC: low and middle-income countries; BE: Belgium; HIC: high-income countries (except BE)

The time course of AM BC in each of the 45 individual subjects is displayed in Additional file 1: Figure S1. The DIC of the models without and with consideration of the group effect amounted to − 43.6 and − 202.91, respectively. The difference of 159.31 between these DIC values indicates that the model that takes into account the group to which individuals belong has a better fit than the model that does not allow for group differences. Figure 2 depicts the time course of AM BC for the three groups, with their posterior predictive checks. The estimated parameters are summarized in Table 2 and graphical presentations of the density distributions are shown in Additional file 1: Figure S2. The LMIC group exhibited a significantly higher initial quantity (R_0_) of median AM BC (1.12 μm^2^) than the HIC group (0.39 μm^2^) and the BE group (0.29 μm^2^). The HIC group had a higher initial quantity of AM BC than the BE group, but with considerable overlap between the two groups. Neither in the HIC group nor the BE group did the decay constants (k) differ from zero. In contrast, the LMIC group exhibited a significant decline in median AM BC with time (with an estimated k = 0.013 μm^2^/day), reaching the values observed among the HIC group after about approximately three months. Thus, based on the exponential decay model, a mean value of 53 days (95% credible interval: 30 to 99 days) was obtained for the clearance half-life of AM BC in the LMIC group.

**Table 2:** Kinetic parameters of median value of AM BC in three groups of participants

Parameter	LMIC group	HIC group	BE group
N	15	15	15
R_0_, μm^2^	1.122 ± 0.193 (0.750–1.509)	0.387 ± 0.110 (0.168–0.613)	0.275 ± 0.066 (0.147–0.404)
k, μm^2^/day	0.013 ± 0.004 (0.007–0.023)	0.002 ± 0.001 (−0.001–0.004)	−0.001 ± 0.000 (− 0.001–0.000)

Data are posterior mean ± SD and 95% credible intervals. *N* number of participants, *R*_*0*_ initial median value of AM BC, *k* decay constant, *LMIC* low and middle-income countries, *HIC* high-income countries, *BE* Belgium

After correcting for gender, age, and season, the DIC values (Additional file 1: Table S3) did not suggest that any of the covariate corrections resulted in a better fitting model. When interpreting the DIC values very liberally, the age-corrected model seemed slightly better, however, the parameter estimates for the age effect on R_0_ and k did not differ from zero (Additional file 1: Table S4).

When the 90th percentile values of AM BC were considered instead of the median values, qualitatively similar findings were obtained (Table 3, Additional file 1: Figure S3). The starting levels were higher for the LMIC group than for the two other groups, which did not differ significantly from each other. However, in the LMIC group, the decay appeared to be slower for the 90th percentile value of AM BC than for the median value (see Additional file 1: Figure S4 for graphical presentation). Hence, the mean (95% credible interval) half-life was 116 days (63 to 231 days) when the 90th percentile value was considered.

**Table 3:** Kinetic parameters of 90th percentile value of median AM BC in three groups of participants

Parameter	LMIC group	HIC group	BE group
N	15	15	15
R_0_, μm^2^	5.809 ± 1.197 (3.555–8.257)	2.683 ± 0.556 (1.613–3.793)	2.238 ± 0.451 (1.358–3.182)
k, μm^2^/day	0.006 ± 0.002 (0.003–0.011)	0.002 ± 0.001 (0.000–0.003)	0.000 ± 0.001 (− 0.001–0.002)

Data are posterior mean ± SD and 95% credible intervals. *N* number of participants, *R*_*0*_ initial 90th percentile value of AM BC value, *k* decay constant, *LMIC* low and middle-income countries, *HIC* high-income countries, *BE* Belgium

#### The evolution of AM BC for sub-groups

##### BE*native* versus BE*foreign*

Within the group of long-term Belgian residents (Table 4), the DIC value for the model taking sub-group differences (natives vs foreign-born) into account was − 173.37, and very close to the DIC value (− 164.31) obtained without considering sub-group differences, thus suggesting that the differences between these two sub-groups were not significant. The foreign-born residents seemed to have twice as much AM BC than the native Belgians (0.37 μm^2^ vs 0.18 μm^2^), but the overlap between the group was high (Table 4). The decay constants were similar in both subgroups and they did not differ from zero (Table 4) (see Additional file 1: Figures S5 and S6 for graphic presentation).

**Table 4:** Kinetic parameters of median value of AM BC in subgroups

Group	N	R_0_, μm^2^	k, μm^2^/day
BE			
BE*native*	7	0.176 ± 0.049 (0.080–0.279)	0.000 ± 0.001 (− 0.002–0.002)
BE*foreign*	8	0.371 ± 0.122 (0.138–0.627)	−0.001 ± 0.001 (− 0.002–0.000)
HIC			
HIC*moderate*	11	0.469 ± 0.167 (0.151–0.816)	0.003 ± 0.002 (0.001–0.008)
HIC*low*	4	0.264 ± 0.126 (0.052–0.549)	−0.002 ± 0.003 (− 0.005–0.002)

Data are posterior mean ± SD and 95% credible intervals. *N* number of participants, *R*_*0*_ initial median value of AM BC, *k* decay constant, *BE* Belgium, *HIC* high-income countries

##### HIC*moderate* versus HIC*low*

Within the group of subjects who had recently arrived in Belgium from other high-income countries (Table 4), the DIC value for the model taking into account sub-group differences (moderate versus low PM exposure) was − 82.05, and similar to the DIC value without consideration of sub-group differences (− 81.8), indicating that the differences between these two sub-groups were not significant. Nevertheless, the HIC*low* sub-group tended to have a lower value for initial AM BC than the HIC*moderate* sub-group (0.26 μm^2^ vs 0.47 μm^2^). Moreover, the positive decay constant in the HIC*moderate* sub-group indicated that AM BC decreased significantly after arrival in Belgium, whereas the negative decay constant in the HICLOW group suggested that AM BC tended to increase after arrival in Belgium (Table 4) (see Additional file 1: Figures S7 and S8 for graphic presentation).

## Discussion

Our quasi-experimental study provides important novel information to evaluate the validity of using carbon load in airway macrophages as a biomarker of chronic exposure to combustion-related particles. The inclusion, for the first time in the same study, of persons originating from countries with different degrees of ambient pollution allowed us to compare the biomarker at an international level. Moreover, making multiple measurements over almost one year in the same individuals allowed us to investigate the time course of AM BC in stable and changing exposure conditions. Although the number of subjects (*n* = 45) included in our study may appear low, the total number of measurements obtained (*n* = 321) was much higher than in previous publications [6]. Our dataset combined high temporal and spatial variation within the same person and, therefore, allowed us to calculate the kinetics of the clearance of inhaled pollutant particles of young healthy subjects.

In brief, we showed that AM BC remained stable in young, healthy, nonsmoking subjects who resided in an area of moderate air pollution during one year. In contrast, AM BC decreased in subjects who moved from an area of high air pollution to an area of moderate pollution.

### Interpretation of the present findings

To our knowledge, no studies have been published in which AM BC has been measured repeatedly in the same subjects over a period of several months. In the group of Belgian residents, whose exposure to air pollution remained stable over the course of about one year (PM_10_ between 20 and 30 μg/m^3^), we observed that, on average, AM BC also remained very stable as reflected in average decay constants (k) of 0 for both the median (Table 2) and the 90th percentile (Table 3) values of AM BC. However, this does not mean that the biomarker did not fluctuate with time within individuals. Thus, among the seven native Belgian volunteers in the BE group the intra-individual variability, expressed as a variation coefficient, ranged from 15% to 78% (average 40%) for the median AM BC, and from 18% to 87% (average 40%) for the 90th percentile value. This variability is probably attributable to inherent imprecisions of the measurement and day-to-day variations in sampling efficiency of sputum, but part of the variability could conceivably also be due to real short-term variations in pollutant exposure, but we did not investigate this aspect further.

We did, however, attempt to find factors that could explain differences in AM BC between individuals. Factors such as sex and age did not appear to be important, at least not within this relatively small group of young healthy subjects. In a study of children, those with asthma had lower AM BC than those without asthma [16], but this is unlikely to have played a role in our study because only few subjects (6/45) had a history of mild asthma. The only significant factor proved to be the geographical origin.

Among the group of Leuven residents, the eight foreign-born participants exhibited about two-fold higher values of AM BC (R_0_ = 0.37 μm^2^) than the seven native Belgians (R_0_ = 0.18 μm^2^). It is unlikely that the difference was due to genetic factors. Kulkarni et al. [16] reported that non-white children had higher AM BC than white children but this could be attributed to a higher residential PM_10_ in non-white children than white children. In our study, all the participants lived and studied or worked as researchers in the Leuven area. Although some variation in residential exposure could have occurred, we do not think that current higher residential PM could explain the observed higher AM BC in the foreign-born control participants compared to the native Belgians. A more plausible explanation is that although these foreign-born subjects had resided in Belgium for a median of 1.9 years (range 1 to 5.4 years), they all had previously lived in areas of higher air pollution than Leuven. We speculate that the higher (and apparently stable) AM BC of these foreign-born residents was due to their higher exposure to combustion-related air pollution in a distant past. In other words, their “baseline” values possibly still reflected a higher lung burden of combustion-related particles.

The participants who had recently arrived in Belgium from cities with high ambient pollution (annual PM_10_ > 50 μg/m^3^) in low and middle-income countries (mainly China and India), exhibited a markedly higher median AM BC at the start of the observation (R_0_ = 1.12 μm^2^) than the control group (R_0_ = 0.28 μm^2^). Their values decreased with an estimated initial half-life of 53 days to reach, after about two half-lives, an apparent steady state at a level marginally higher than the controls. The 90th percentile values of AM BC were also higher, but they decreased at a much slower rate (initial half-life of 116 days).

Overall, the initial median AM BC level of the participants who had recently arrived from high-income countries (HIC group), was a little higher (R_0_ = 0.39 μm^2^) than that of the control group of long-term Leuven residents (R_0_ = 0.28 μm^2^) and it remained constant at that somewhat higher level throughout the observation period. Nevertheless, when this heterogeneous group was subdivided into two subgroups according to the annual level of PM_10_ exposure prior to arriving in Leuven, a consistent though not always statistically significant pattern emerged. Past exposure below the Leuven level (HIC*low*, 4 subjects) was associated with a lower estimated initial AM BC (R_0_ = 0.26 μm^2^). In contrast, past exposure above or similar to the Leuven level (HIC*moderate*, 11 subjects) was associated with a somewhat higher initial AM BC (R_0_ = 0.47 μm^2^) and a slow but significant decay (k = 0.003 μm^2^/day). This slow decrease is compatible with our hypothesis proposed above to explain the higher median AM BC in the foreign-born Leuven residents compared to the native Belgians.

### Clearance kinetics of particles from the lungs

Although the deposition and fate of inhaled particles in the respiratory tract has been extensively studied [17–19], surprisingly little knowledge is available about the clearance kinetics of pollutant particles that have deposited in the lungs of healthy subjects.

We had hoped to observe an increase in AM BC in people arriving in Leuven from areas of lower pollution. However, we could recruit only four such subjects, thus precluding any firm conclusion about the kinetics of AM BC following an increase in exposure. Nevertheless, our findings in the small HIC*low* group are suggestive of a rapid increase in median AM BC after arrival in Leuven (from 0.16 μm^2^ at T1 to 0.33 μm^2^ at T2); this early increase was not reflected in the Bayesian analysis because this analysis considered the entire observation period.

However, we did find that moving from high pollution to moderate pollution was followed by a progressive decrease in AM BC. It should be realized that in our real-life study, the participants who had left areas of high urban pollution did not arrive in an area devoid of exposure, but in a city where they still inhaled pollutant particles. In other words, the observed kinetics represent the *net* result of clearance and a continued (albeit lower) deposition of combustion particles. In these circumstances, the AM BC clearance half-life was 53 days, when taking the median value of AM BC, and 116 days when taking the 90th percentile value. Both estimates are shorter than the half-lives suggested in prior studies [17]. There are several possible explanations for this discrepancy (apart from technical factors and computational differences). A probably important factor relates to the fact that we based our conclusions on particle load in AMs obtained by sputum induction, which retrieves AMs from central and larger airways rather than from distal bronchioles and alveoli [6]. In other words, the particles studied in our study had already migrated from the alveoli to the conducting ciliated airways, where clearance half-lives between 5 and 30 days have been described [17]. Another, possibly important factor concerns the exposure level, which was not thoroughly investigated in previous studies. In our study, the highly exposed participants came from areas where annual average PM_10_ varied from 55 to 171 μg/m^3^ (Additional file 1: Table S2), according to the WHO database [11]. This real-life exposure level is lower than experimental exposure conditions [20]. Finally, the physicochemical properties of inhaled particles can significantly influence the deposition, retention, and clearance of inhaled particles [17, 18, 20, 21]. The majority of the previous kinetic studies that used animals with exposure to radiolabeled or fluorescently labelled particles in laboratory conditions generated a wide range of clearance half-lives, from a few tens to several hundred days [17]. To our knowledge, kinetic studies following long-term exposure to environmental PM in humans have not been reported. Of note, in a lung-transplantation setting, the donor’s AMs were shown to persist for up to 2 to 3.5 years [22, 23], implying that the replacement of AMs can be prolonged up to a few years, which means that the clearance of environmental PM through AMs is a slow process.

When we used 90th percentile values of AM BC, the calculated half-life was considerably longer (116 days). Moreover, R_0_ for the 90th percentile values of AM BC was approximately five-fold higher than the median value of AM BC. The prolonged clearance half-life calculated with the 90th percentile values of AM BC suggests that it takes longer to clear particle-overloaded lungs.

### Strengths and limitations

Using carbon load in airway macrophages to assess past exposure to ambient pollutant particles is critically dependent on the availability of adequate sputum samples, which may be problematic even with the technique of sputum induction. In the present study, four out of 50 volunteers proved unable to expectorate on any of the eight scheduled visits, and some subjects occasionally failed, for unclear reasons, to provide an adequate sample, thus resulting in an overall success rate of 85% for the sample collection. Nevertheless, this success rate was higher than in previous studies [7, 14, 16], and allowed us to study the kinetic behavior of AM BC with a relatively low number of participants.

A critical issue concerns the basic assumption that the area occupied by black inclusions detected by light microscopy in macrophages reflects the amount of elemental carbon in the cells. In 2001, Bunn et al. [24] showed that black particles in AMs were indeed composed of carbon, but none of the studies that have since used image analysis to quantify AM BC have attempted to document the chemical composition of the black particles [6]. Although the primary purpose of the present study was not to verify the carbonaceous nature of the black inclusions in AM, we provided preliminary data, illustrated in Fig. 1, showing that our quantification method of AM BC correlated well with an independent specific detection and quantification of carbon engulfed in cells. This novel method is based on the property of carbon particles to emit white light under femtosecond pulsed near-infrared illumination [15] and it has been used to show the presence of carbon particles in urine [25].

## Conclusions

By repeatedly measuring personal AM BC content in young healthy participants having various exposure histories, we were able to describe the temporal evolution of AM BC content. Our participants coming from high pollution areas exhibited significantly higher AM BC content than Belgian residents and participants coming from less polluted areas. Thereafter, the AM BC content from the most polluted group reached the same level as the Belgians in about 100 days, i.e. after two clearance half-lives of 53 days. These findings validate the use of AM BC as a biomarker of long-term exposure to environmental air pollution.

## Additional file

Additional file 1: Detailed methods, additional tables (Tables S1-S4) and additional figures (Figures S1-S8). (DOCX 1541 kb)

## Abbreviations

2D: Two-dimensional
3D: Three-dimensional
AM BC: Carbon load in airway macrophages
AM(s): Airway macrophage(s)
BC: Black carbon
BE: Belgium
DIC: Deviance information criterion
FEV_1_: Forced expiratory volume in 1 s
FVC: Forced vital capacity
HIC: High-income countries
IQR: Interquartile range
k: Decay constant
LMIC: Low and middle-income countries
PM: Particulate matter
PM_10_: Particulate matter in aerodynamic diameter ≤ 10 μm
R_0_: Initial quantity
SD: Standard deviation
