# Supplementary material for: Carbon load in airway macrophages as a biomarker of exposure to particulate air pollution; a longitudinal study of an international Panel
Source: Part Fibre Toxicol. 2018 Mar 14;15:14. doi: 10.1186/s12989-018-0250-8 (PMC5853150; doi:10.1186/s12989-018-0250-8)
Supplement: Supplementary file 1 — Detailed methods, additional tables (Tables S1-S4) and additional figures (Figures S1-S8). (DOCX 1541 kb) [file 12989_2018_250_MOESM1_ESM.docx]

**ADDITIONAL FILE 1**

**Carbon load in airway macrophages as a biomarker of exposure to particulate air pollution. A longitudinal study of an international panel**

Yang Bai ^1^

Email: yang.bai@kuleuven.be

Hannelore Bové ^2, 3^

Email: Hannelore.bove@uhasselt.be

Tim S. Nawrot ^1, 4^

Email: tim.nawrot@uhasselt.be

Benoit Nemery ^1^ *

* Corresponding author

Email: ben.nemery@kuleuven.be

^1^ Environment and Health, KU Leuven, Herestraat 49, O&N 1, box 706, 3000 Leuven, Belgium

² Biomedical Research Institute, Hasselt University, Agoralaan Building C, 3590 Diepenbeek, Belgium

^3^ Center for Surface Chemistry and Catalysis, KU Leuven, Celestijnenlaan 200F, 3001 Leuven, Belgium

^4^ Centre for Environmental Sciences, Hasselt University, Agoralaan Building D, 3590 Diepenbeek, Belgium

**Supplemental methods**

**Induced sputum**

Nebulized saline (3, 4, and 5%) was administered through De Vilbiss nebuliser (Ultra-Neb 2000 model 200HI) in 3 sequential 7-minute inhalation periods. Lung function (spirometry) was measured before each inhalation period for the detection of clinically significant bronchoconstriction. Induced sputum was processed within 2 hours after induction by a modification of the technique described by Pizzichini et al. [1]. Briefly, the plugs of airway cells were selected, weighed and treated with a volume of Hank’s balanced salt solution containing 0.1% dithiothreitol (Sigma, St Louis, MO, USA) and 3% bovine serum albumin (Sigma) of four times the weight was added. Portions were agitated with a vortex, placed on a bench rocker for 5 minutes, filtered through a 70 μm Falcon cell strainer, and centrifuged at 1500 rpm for 10 minutes. The sputum supernatant was removed and stored at -80 ˚C for cytokine analysis. The cell pellet was resuspended in 1000 μl phosphate-buffered saline. A total nonsquamous cell count was performed in a hemocytometer and expressed as millions per milliliter of selected induced sputum. The proportion of salivary squamous cells was noted and cell viability was determined by trypan blue exclusion method. Cytospins were prepared by cytocentrifuging (Shandon Scientific, Techgen, Zellik, Belgium) 15000 cells onto glass slides and stained with Diff-Quik (Medion Diagnostics, Düdingen, Germany).

**Carbon kinetic study models**

In general, the exponential decay model used for the AM BC profiles is given by:

$$y_{i} \sim N\left( R_{0j\left[ i \right]}\exp\left( -\lambda_{j\left[ i \right]}d_{ik} \right), \sigma_{y}^{2} \right),\text{ for} i=1, \ldots, n$$

$$R_{0j} \sim N\left( r_{0}, \sigma_{r0}^{2} \right), \text{for} j=1, \ldots, J$$

$$\lambda_{j} \sim N\left( \gamma_{1}, \sigma_{\gamma}^{2} \right),\text{ for} j=1, \ldots, J$$

Where y_i_ is a vector of all AM BC values measured across all subjects and time, hence $n=J(K)$ where $J$ refers to the number of subjects in the study and $K$ to the number of measurement moments (without taking missing values into account). These AM BC values are assumed to be normally distributed with a mean given by $(R_{0j}\exp\left( -\lambda_{j}d_{ik} \right))$ and a variance of $\sigma_{y}^{2}$. The variable $d$ indicates the days between the first measurement and/or time of arrival and the other measurement moments. The first unknown parameter in the model is called the initial quantity $R_{0j}$ and is the estimated AM BC value at day 0. When the time of arrival and the first measurement did not coincide, a model-based extrapolation for the value at day 0 is made. This initial quantity is important because the estimated decay per day is proportional to this value. This subject-specific initial quantity $R_{0j}$ is drawn from a normal distribution with a mean $r_{0}$ and a variance $\sigma_{r0}^{2}$. More concrete, $R_{0j}$ is the estimated initial quantity for subject $j$ and $r_{0}$ is the estimated initial quantity for a mean subject. The second unknown parameter is called the decay constant and is given by $\lambda_{j}$. The $j$ again refers to the hierarchical nature of the parameter, meaning that this parameter is subject-specific. This decay constant allows the response strength to decay exponentially with increasing time between day 0 and the subsequent measurements. This $\lambda_{j}$ is drawn from a normal distribution with mean $\gamma_{1}$ and variance $\sigma_{\gamma}^{2}$. More concrete, $\lambda_{j}$ is the estimated initial quantity for subject $j$ and $\lambda$ is the estimated initial quantity for a mean subject.

Two models were compared to test for the group effect. The first model did not take any group differences into account. The second model fitted the above-mentioned model in the three groups separately. This latter model resulted in separate fixed, variance, and residual parameters. The Deviance Information Criterion (DIC) is used to make relative comparisons. The DIC can be used to compare the fit of a restricted model (i.e., without group information) that is nested within a full model (i.e., a model that takes the group differences into account). The DIC is a measure of how well the model fits the data, with larger values indicating a worse fit compared to lower values. We also made use of posterior predictive checks to test if the model fits the data well. A model is a statistical representation of the phenomenon on which the data was collected. Simply put, if the model has a good fit, the data that is generated from the model should be similar to the data that was observed. Posterior predictive model checks are concerned with especially this comparison: To what extent is the generated data the same as the observed data? Systematic differences between both data sources can indicate a misspecification or failings of the model. In practice, posterior predictive model checks are achieved by drawing simulated values from the joint posterior distribution of the model and comparing these values with the observed data. Inferences based on parameters itself were done by means of the 95% credible interval. Credible intervals of a parameter that do not contain 0 do differ significantly from 0. The comparison of two parameters can also be done based on these credible intervals: non-overlapping 95% credible intervals mean that both parameters differ from each other. The Bayesian models were estimated with the use of (r)jags [2]. Bayesian parameter estimates need a specification of the prior distribution. For these models, only non-informative priors were used. The fixed effects were drawn from a non-informative normal distribution and the variance components from a non-informative uniform distribution. All models were run in three chains, with 50000 iterations in order that all chains mixed well.

Note: in the output, $\lambda$ is replaced with k.

All analyses were performed using R version 3.3.3 (2017-03-06) in Rstudio.

**Supplemental results**

**Table S1.** Summary of exposures before and after inclusion

|  | **All** | **LMIC** | **HIC** | **BE** |
| --- | --- | --- | --- | --- |
| **N** | 45 | 15 | 15 | 15 |
| **Exposure before inclusion** |  |  |  |  |
| Estimated annual PM_10_, mean µg/m^3^ (SD) # |  | 108 (63 – 171) | 23 (14 – 34) | 23 |
| Passive cigarette smoking, n (%) |  |  |  |  |
| No | 31 (69) | 11 (73) | 8 (53) | 12 (80) |
| At home | 10 (22) | 4 (27) | 4 (27) | 2 (13) |
| At work | 4 (9) | 0 | 3 (20) | 1 (7) |
| Household smoke, n (%) |  |  |  |  |
| No | 36 (82) | 12 (86) ‡ | 12 (86) | 12 (86) |
| Candles/incense | 3 (7) | 0 | 2 (14) | 1 (7) |
| Coal | 3 (7) | 2 (14) | 0 | 1 (7) |
| Wood | 2 (4) | 0 | 1 (7) | 1 (7) |
| Residence environment, n (%) |  |  |  |  |
| Countryside | 9 (21) | 0 ‡ | 2 (13) | 7 (50) ‡ |
| Downtown | 19 (44) | 13 (93) *** | 4 (27) | 2 (14) |
| Suburb | 15 (35) | 1 (7) | 9 (60) | 5 (36) |
| Potential environmental pollution sources, n (%) |  |  |  |  |
| Not identified | 5 (11) | 1 (7) ‡ | 0 | 4 (27) |
| Traffic | 11 (25) | 2 (14) | 9 (60) | 0 |
| Industrial | 10 (23) | 3 (21) | 3 (20) | 4 (27) |
| Traffic and industrial | 18 (41) | 8 (57) | 3 (20) | 7 (47) |
| **Exposure after inclusion** |  |  |  |  |
| Passive cigarette smoking, n (%) |  |  |  |  |
| No | 43 (96) | 15 (100) | 14 (93) | 14 (93) |
| At home | 0 | 0 | 0 | 0 |
| At work | 2 (4) | 0 | 1 (7) | 1 (7) |
| Household smoke, n (%) |  |  |  |  |
| No | 41 (91) | 13 (87) | 13 (87) | 15 (100) |
| Candles/incense | 3 (7) | 1 (7) | 2 (13) | 0 |
| Wood | 1 (2) | 1 (7) | 0 | 0 |

‡ n = 14. # Data are expressed as median (range). Comparison among groups by one-way ANOVA or χ^2^ test. ****p* < 0.001.

**Table S2.** Cities and annual average PM_10_ ^#^

| Group | Country | City | Number of participants | Annual PM_10_, µg/m^3^ |
| --- | --- | --- | --- | --- |
| LMIC | China | Zhengzhou | 1 | 171 |
|  | India | Kolkata | 1 | 135 |
|  | China | Beijing | 6 | 108 |
|  | China | Suqian | 1 | 102 |
|  | China | Suzhou | 1 | 97 |
|  | Peru | Lima | 1 | 88 |
|  | India | Hyderabad | 1 | 79 |
|  | China | Datong | 1 | 63 |
|  | India | Chennai | 1 | 57 |
|  | Brazil | Sao Paulo | 1 | 55 |
|  |  |  |  |  |
| HIC | Italy | Padova | 1 | 34 |
|  | Italy | Verona | 1 | 34 |
|  | Italy | Rome | 2 | 28 |
|  | Croatia | Vukovar | 1 | 27 ^‡^ |
|  | Spain | Barcelona | 2 | 24 |
|  | The Netherlands | Dordrecht | 1 | 24 |
|  | United Kingdom | London | 2 | 22 |
|  | Luxembourg | Luxembourg | 1 | 22 |
|  | Spain | Madrid | 1 | 19 |
|  | Luxembourg | Reutlingen | 1 | 18 |
|  | Ireland | Dublin | 1 | 16 |
|  | Estonia | Tallinn | 1 | 14 |
|  |  |  |  |  |
| BE | Belgium | Leuven | 15 | 23 |

^#^ Data for annual PM_10_ were extracted from the WHO database of urban ambient air pollution [3] for the nearest city. ^‡^ For Croatia, annual PM_10_ value was estimated from the study by Jeričević *et al.* [4].

**Table S3.** Comparison between original model and covariate-corrected models with deviance information criterion values

| **Model** | **DIC** | **Difference to original model** |
| --- | --- | --- |
| Original model without covariates | -202.91 |  |
| Gender corrected | -204.73 | -1.82 |
| Age corrected | -219 | -8.99 |
| Season corrected | -183.89 | 19.02 |

DIC, deviance information criterion. The smaller DIC is, the better fitting the model is.

**Table S4.** Posterior mean ± SD and 95% credible intervals of median AM BC kinetic parameters for groups with age correction #

| Parameter | LMIC group | HIC group | BE group |
| --- | --- | --- | --- |
| R_0_. µm^2^ | 0.053 ± 0.043  (0.002 – 0.157) | 0.040 ± 0.037  (0.001 – 0.135) | 0.035 ± 0.022  (0.002 – 0.086) |
| k. µm^2^/day | -0.002 ± 0.001  (-0.004 – 0.000) | 0.000 ± 0.001  (-0.001 – 0.002) | 0.000 ± 0.000  (0.000 – 0.000) |

# The age-corrected model gave the smallest DIC, suggesting it has a better fit. However, the decay constant for LMIC group did not differ from zero, which is against the original model.

R_0_, initial AM BC value; k, decay constant; LMIC, low middle-income countries; HIC, high-income countries; BE, Belgium.

**Figure S1.** AM BC profiles with posterior predictive checks for individual level

**
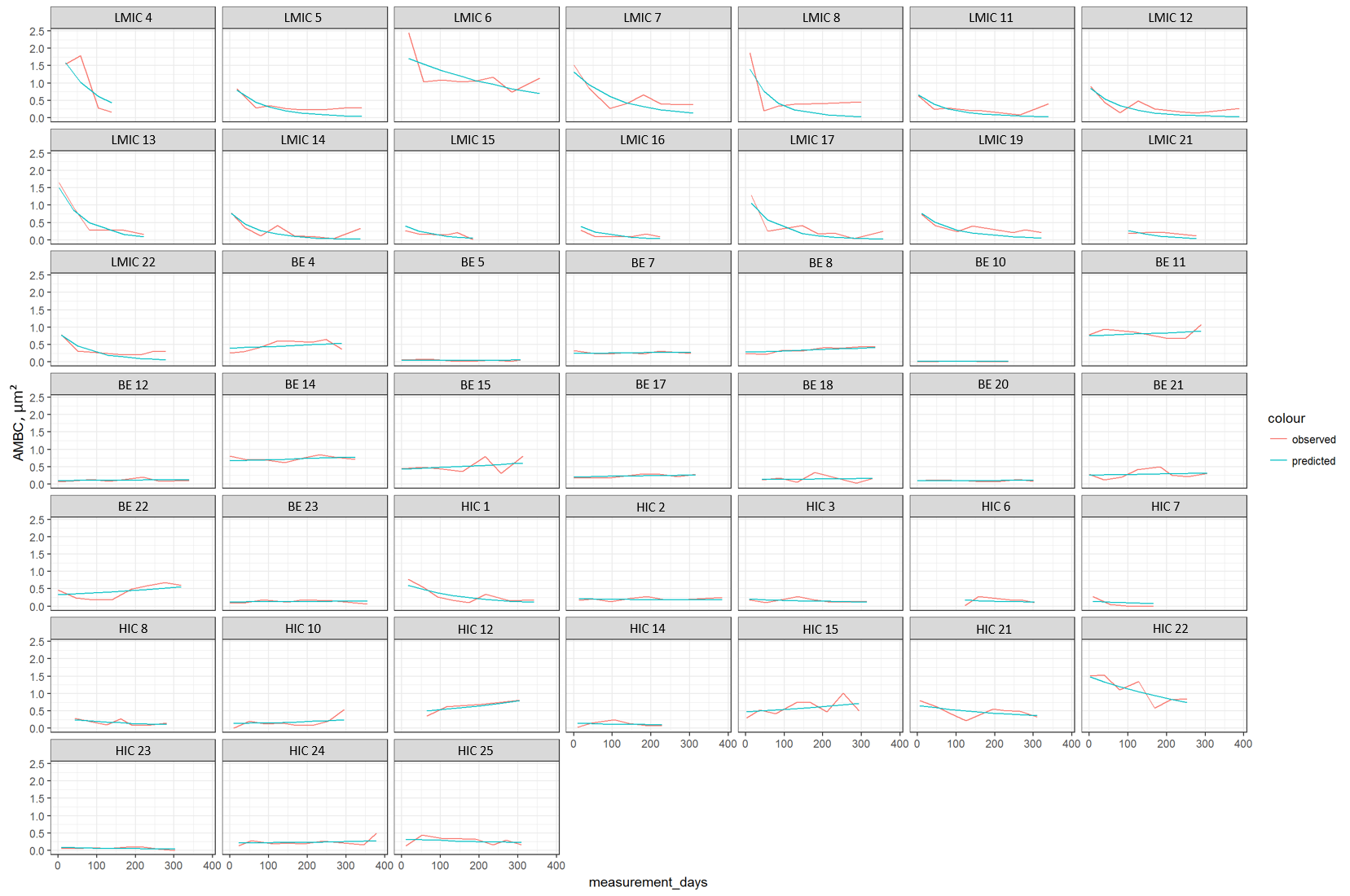
**

Observed subject-specific AM BC profiles (red lines) versus model predicted subject-specific AM BC profiles (blue lines). The model allows for a group specific exponential decay of AM BC values over the days after the initial measurement (for local residents) or day of arrival (for newcomers).

**Figure S2.** Density plots for the group specific variance estimates for the initial quantity of median AM BC (R_0_) and decay constant (k)


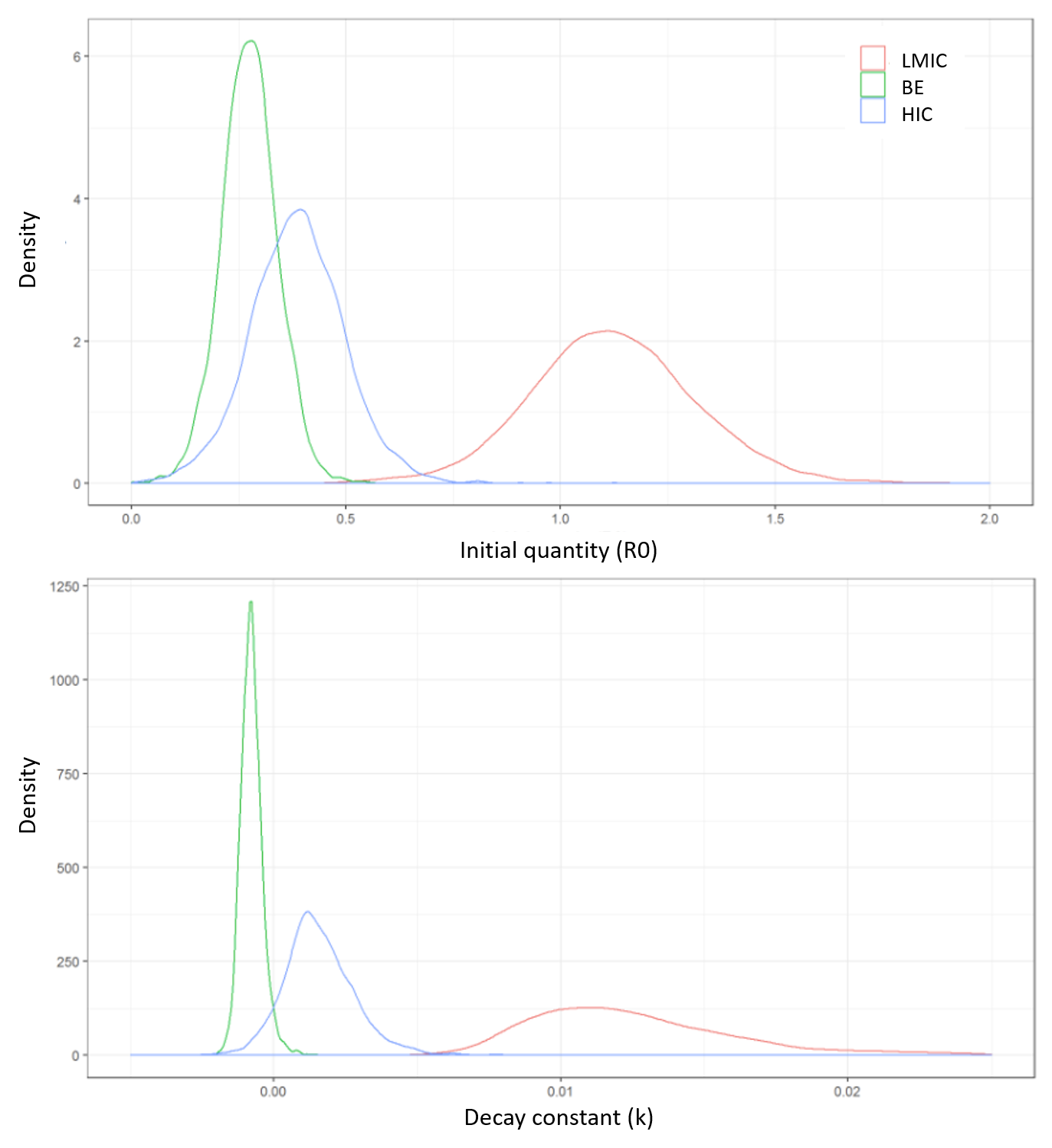


LMIC, low and middle-income countries; BE, Belgium; HIC, high-income countries.

The density plots allow an evaluation to be made of the degree of overlap between groups.

**Figure S3.** 90^th^ percentile values of AM BC with posterior predictive check for groups


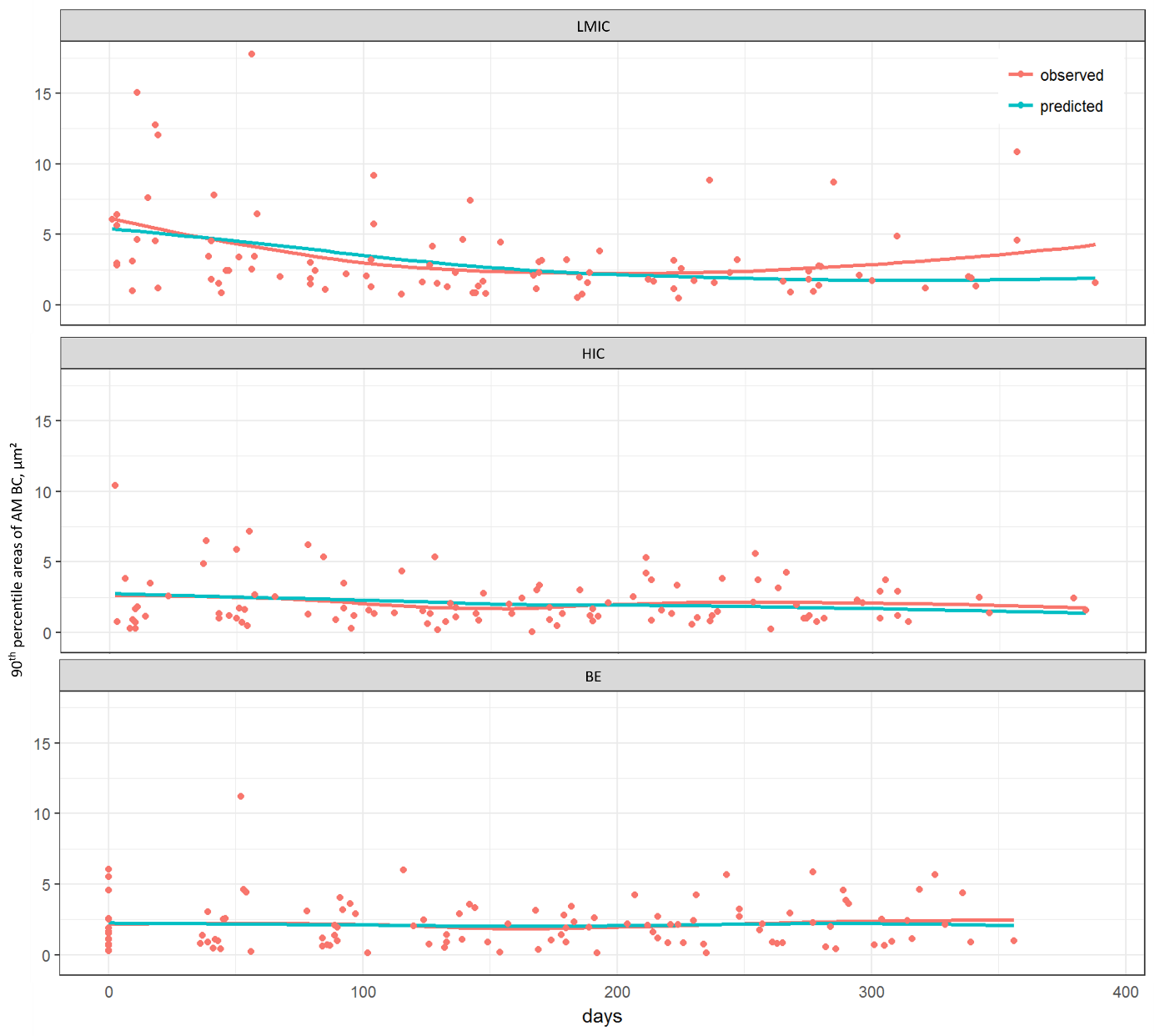


Observed loess-smoothed AM BC profiles (red lines) versus model predicted loess-smoothed AM BC profiles (blue lines). Each dot represents the 90^th^ percentile value of AM BC obtained from one participant at one time point (15 participants in each group). LMIC: low and middle-income countries; BE: Belgium; HIC: high-income countries (except BE).

**Figure S4.** Density plots for the group specific variance estimates for the initial quantity (R_0_) and decay constant (k) for the 90^th^ percentile AM BC values
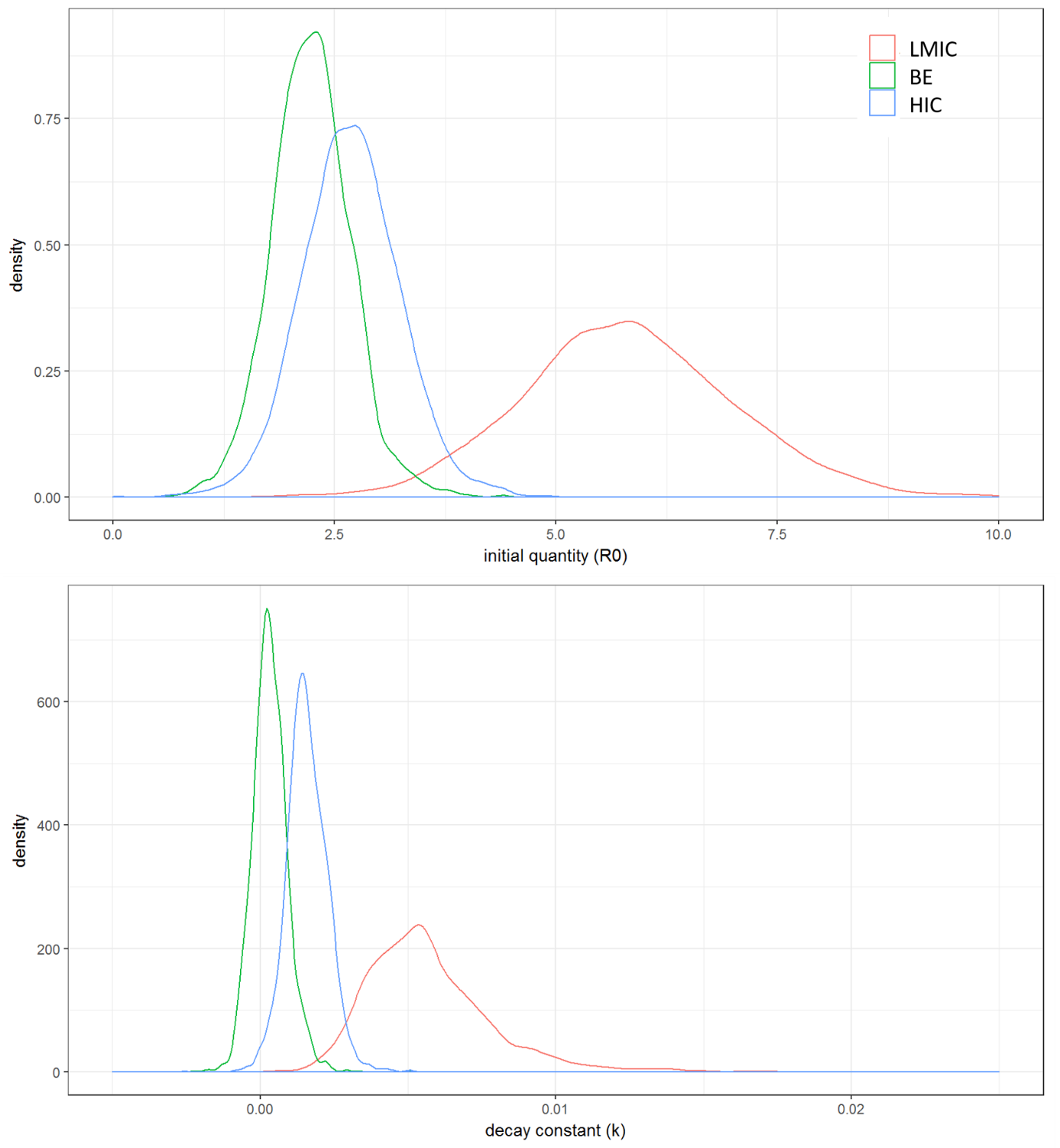


LMIC, low and middle-income countries; BE, Belgium; HIC, high-income countries.

**Figure S5.** AM BC profiles with posterior predictive check for subgroups among Belgian residents (foreign-born vs native)
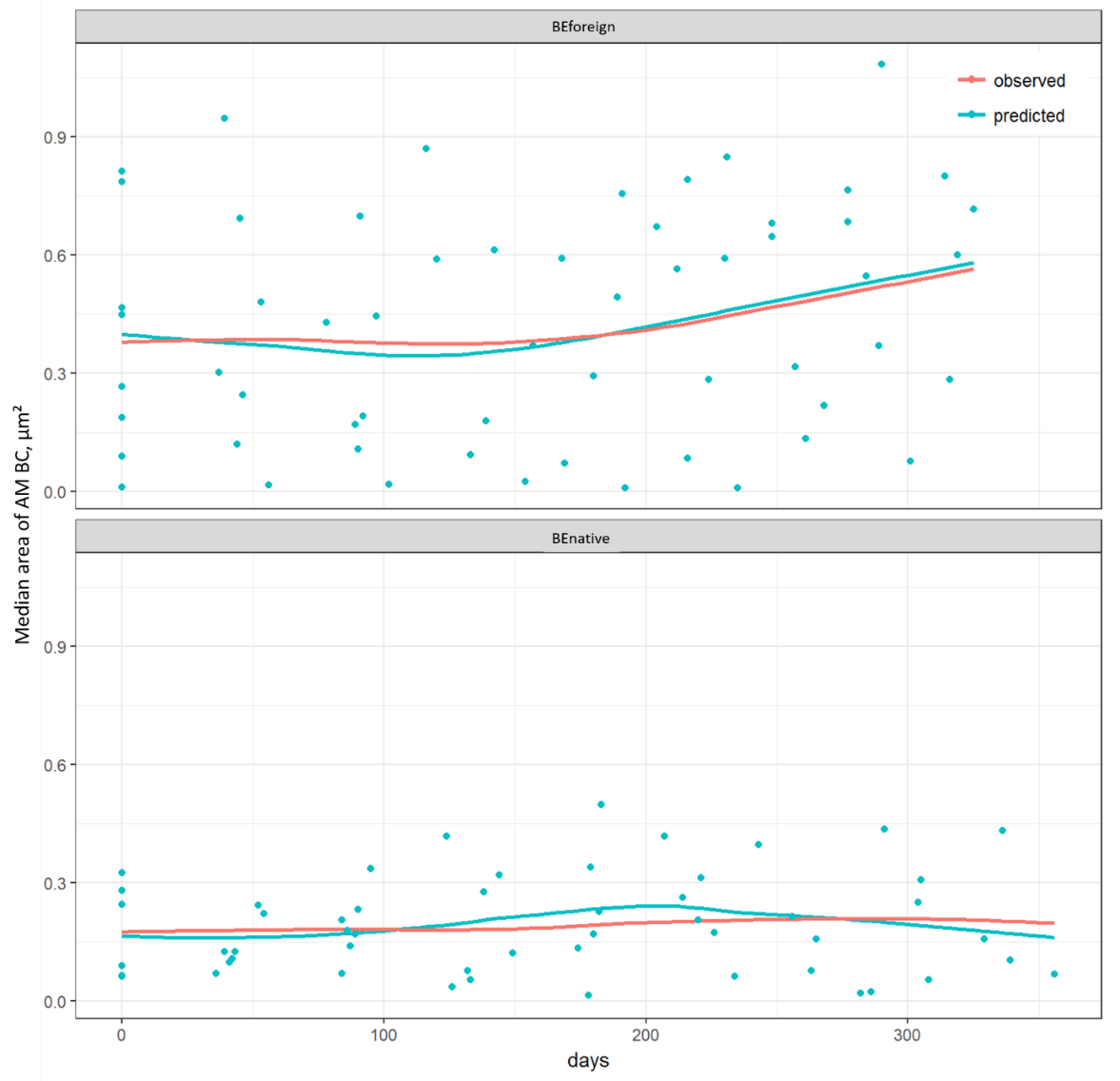


Observed loess-smoothed AM BC profiles (red lines) versus model predicted loess-smoothed AM BC profiles (blue lines). Each dot represents a median value of AM BC obtained from one participant at one time point.

**Figure S6.** Density plots for the subgroup specific variance estimates for the initial quantity (R_0_) and decay constant (k) in Belgian residents (foreign-born vs native) for median AM BC values
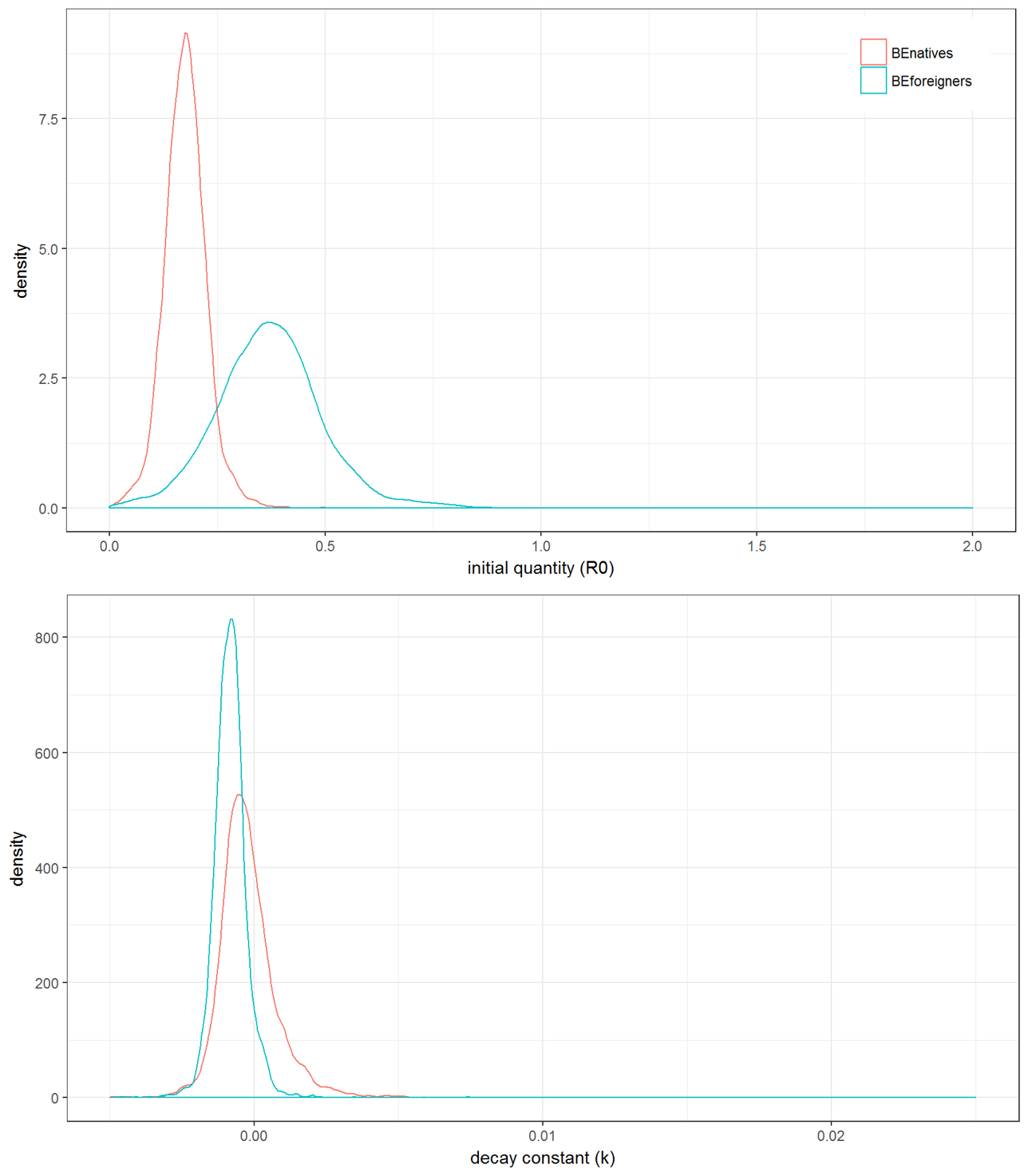


**Figure S7.** AM BC profiles with posterior predictive check for subgroups among high-income countries (HIC*LOW* vs HIC*MODERATE*)
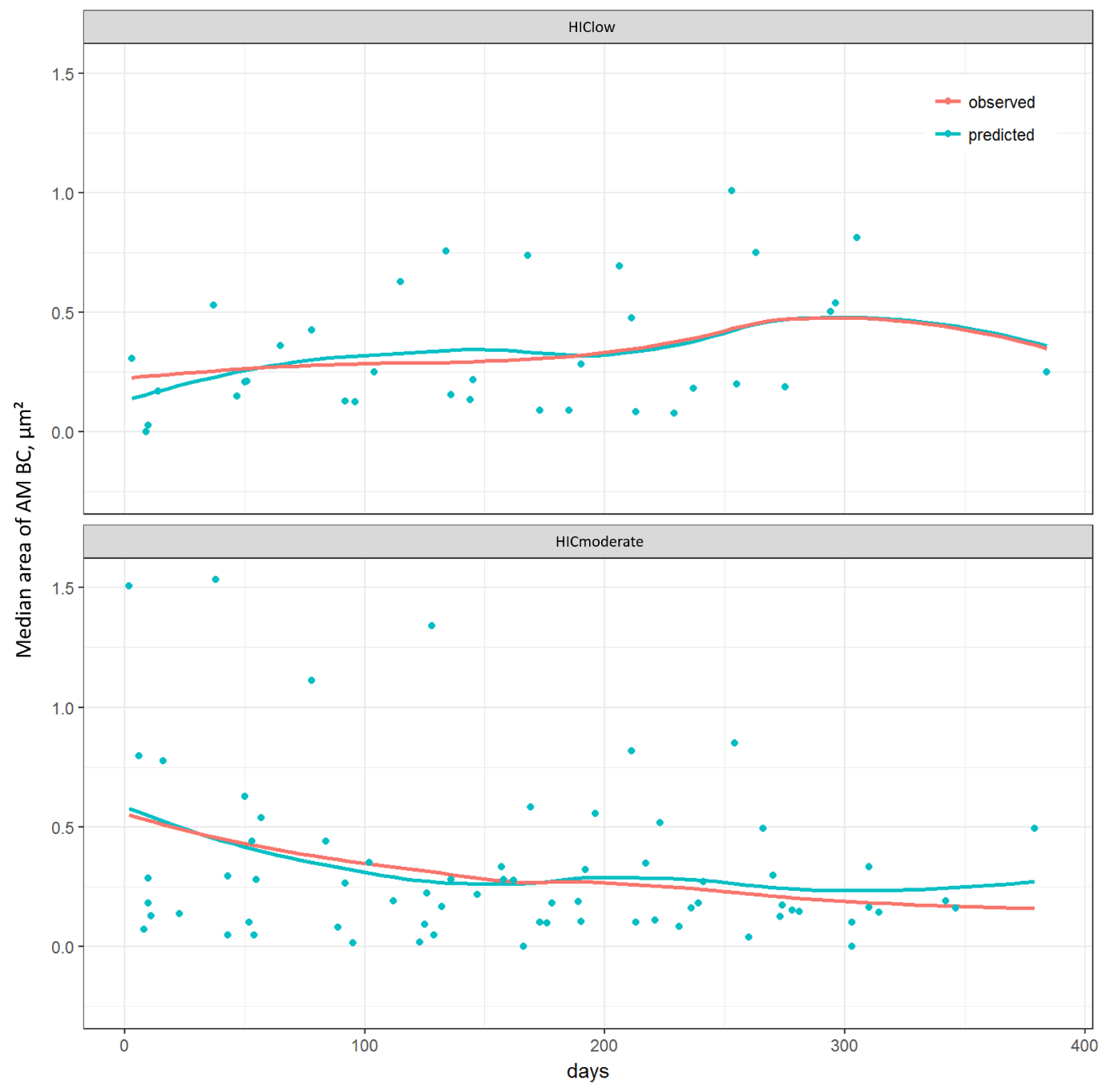


Observed loess-smoothed AM BC profiles (red lines) versus model predicted loess-smoothed AM BC profiles (blue lines). Each dot represents a median value of AM BC obtained from one participant at one time point.

**Figure S8.** Density plots for the subgroup specific variance estimates for the initial quantity (R_0_) and decay constant (k) in high-income countries (HIC) group (HICLOW vs HICMODERATE) for median AM BC


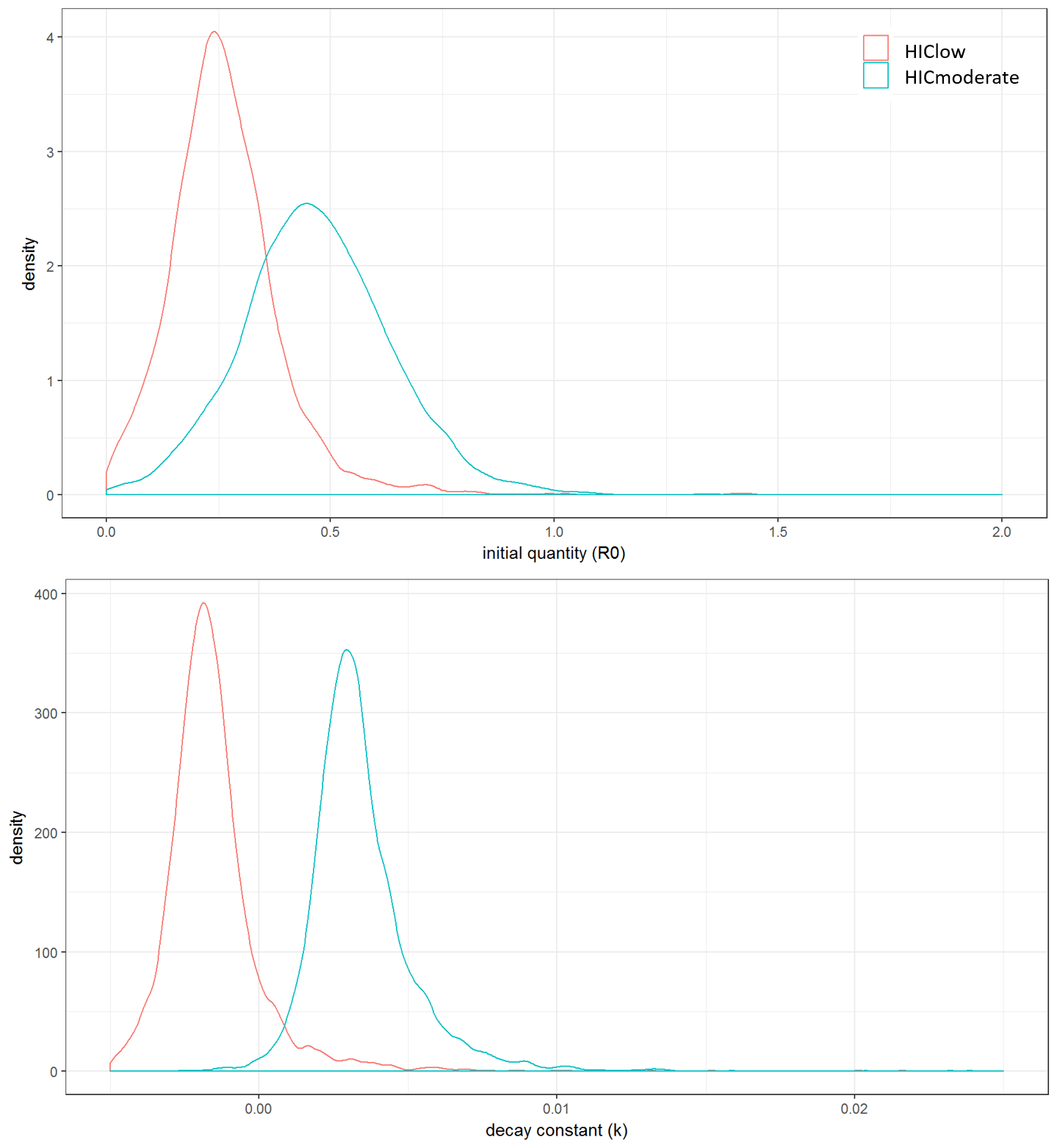


**Supplemental references**

1. Pizzichini E, Pizzichini MM, Efthimiadis A, Evans S, Morris MM, Squillace D, et al. Indices of airway inflammation in induced sputum: reproducibility and validity of cell and fluid-phase measurements. Am. J. Respir. Crit. Care Med. 1996;154:308–17.

2. Plummer M. DSC 2003 Working Papers JAGS: A program for analysis of Bayesian graphical models using Gibbs sampling.

3. World Health Organization. WHO Global Urban Ambient Air Pollution Database (update 2016). 2016. http://www.who.int/phe/health_topics/outdoorair/databases/cities/en/. Accessed 13 Dec 2017.

4. Jeričević A, Džaja Grgičin V, Telišman Prtenjak M, Vidič S, Bloemen H. Analyses of urban and rural particulate matter mass concentrations in Croatia in the period 2006-2014. Geofizika. Andrija Mohorovicic Geophysical Institute; 2016;33:157–81.
